# Quantification of Hepatic Vascular and Parenchymal Regeneration in Mice

**DOI:** 10.1371/journal.pone.0160581

**Published:** 2016-08-05

**Authors:** Chichi Xie, Lars Ole Schwen, Weiwei Wei, Andrea Schenk, Sara Zafarnia, Felix Gremse, Uta Dahmen

**Affiliations:** 1 Department of General, Visceral and Vascular Surgery, Jena University Hospital, Jena, Thüringen, Germany; 2 Fraunhofer Institute for Medical Image Computing MEVIS, Bremen, Bremen, Germany; 3 Institute for Experimental Molecular Imaging, RWTH Aachen University, Aachen, Nordrhein-Westfalen, Germany; University of Bari Medical School, ITALY

## Abstract

**Background:**

Liver regeneration consists of cellular proliferation leading to parenchymal and vascular growth. This study complements previous studies on cellular proliferation and weight recovery by (1) quantitatively describing parenchymal and vascular regeneration, and (2) determining their relationship. Both together are needed to (3) characterize the underlying growth pattern.

**Methods:**

Specimens were created by injecting a polymerizing contrast agent in either portal or hepatic vein in normal or regenerating livers after 70% partial hepatectomy. 3D image data were obtained through micro-CT scanning. Parenchymal growth was assessed by determining weight and volume of the regenerating liver. Vascular growth was described by manually determined circumscribed parameters (maximal vessel length and radius of right inferior portal/hepatic vein), automatically determined cumulative parameters (total edge length and total vascular volume), and parameters describing vascular density (total edge length/volume, vascular volume fraction). The growth pattern was explored by comparing the relative increase of these parameters to the increase expected in case of isotropic expansion.

**Results:**

Liver volume recovery paralleled weight recovery and reached 90% of the original liver volume within 7 days. Comparing radius-related vascular parameters immediately after surgical resection and after virtual resection in-silico revealed a slight increase, possibly reflecting the effect of resection-induced portal hyperperfusion. Comparing length-related parameters between post-operative day 7 and after virtual resection showed similar vascular growth in both vascular systems investigated. In contrast, radius-related parameters increased slightly more in the portal vein. Despite the seemingly homogeneous 3D growth, the observed vascular parameters were not compatible with the hypothesis of isotropic expansion of liver parenchyma and vascular structures.

**Conclusion:**

We present an approach for the quantitative analysis of the vascular systems of regenerating mouse livers. We applied this technique for assessing the hepatic growth pattern. Prospectively, this approach can be used to investigate hepatic vascular regeneration under different conditions.

## Introduction

Livers have the remarkable capability to fully regenerate after major loss of parenchyma. Regeneration requires reconstitution of liver parenchyma and vascular structures. Two major cell types involved in the regeneration at a microscopic scale are hepatocytes and liver sinusoidal endothelial cells (LSECs). Proliferation of these cells leads to the increase of liver mass and growth of blood vessels, respectively [1]. The present study focuses on these changes on a meso- and macroscopic scale.

In the past, more efforts were spent on studying parenchymal liver regeneration rather than on vascular regeneration. Parenchymal growth is typically quantified on the cellular level by determining the hepatocyte proliferation index and on the lobule or organ level by measuring hepatic weight or volume, see [2] for a review.

In particular, changes in geometry and shape of the hepatic lobes are typically not addressed. Inhomogeneous growth of the liver or of a given liver lobe may point to a disturbance in liver regeneration. Thus, we here investigated the growth pattern in liver regeneration. Based on macroscopic observations, one could assume that liver regeneration resembles isotropic expansion. Therefore we wanted to compare the changes in vascular and parenchymal parameters observed after seven days of regeneration to the ones expected in case of isotropic expansion.

Growth and remodeling of liver vessels seems to be crucial in the process of hepatic regeneration. Vascular regeneration mainly consists of the prolongation of the main vessel branches and outgrowth of small terminal branches. Studying vascular regeneration can facilitate the understanding of the pivotal role of vascular growth for the process of regeneration. Traditionally, vascular growth is assessed on a cellular level indirectly by quantifying proliferation of LSECs with specific markers [3]. However, it is still hard to quantify vascular growth routinely and directly on the macroscopic level.

As the development of imaging techniques, there are several approaches available for visualizing vascular growth [4] on the lobule or organ scale in different organ systems. Silicone injection in combination with micro-CT (μCT) imaging techniques are established and commonly used for assessing vascular growth. This contrasting technique has been used to successfully evaluate vasculature in organs (e.g., brain [5], liver [6] and bone [7;8]) and tumors [9;10]. This technique allows a more thorough structural characterization of vasculature than 2D images. Imaging also provides quantitative data of vascular growth, which allows a mathematical description of the biological phenomenon of regeneration. Despite these promising advances for visualization and quantification of vascular regeneration, this technique was not well established for livers in small experimental animals. We previously adapted this technique to rodent livers [11]. It proved to be very helpful to identify anatomical variants. This study shows that the technique is also useful for quantifying regeneration.

Quantifying vascular growth requires numerically evaluating the changes in parameters appropriate for describing the geometry of vascular systems. Such parameters include vessel diameter/ thickness [6–8], vessel length [12;13], vessel volume [9;14;15], angles at branchings [16], vessel number [10;17], and vessel cross-section area [18]. Parameters are determined for individual vessel segments or as cumulative quantities in the literature mentioned before. Additionally, the dependency on the hierarchy in vascular trees was considered [18–20]. However, there is no widely accepted standard yet to describe vascular geometries.

The goals of the present study were to (1) quantitatively describe the increase of parenchymal and vascular growth during regeneration, and (2) to quantify the relationship between regenerating parenchyma and vasculature in order to (3) compare the observed growth to hypothetical isotropic expansion.

For this purpose, 70% partial hepatectomy (PH) was performed in mice, specimens of the hepatic vasculature at different time points were created by injection of a radiopaque polymerizing agent, μCT imaging was performed, and geometric representations of the vasculature were obtained from the image data. Geometric parameters evaluated in the vascular data sets of normal and liver-resected mice. Determining parameters at different time points provided a quantitative description of vascular regeneration which was, in turn, compared to parenchymal volume recovery to explore the growth pattern. Furthermore, these changes in geometric parameters were compared to changes expected in case of isotropic expansion. This finally led to the conclusion that the observed regeneration did not resemble isotropic expansion.

## Material and Methods

### Experimental Design—Workflow Overview

**Fig 1:**
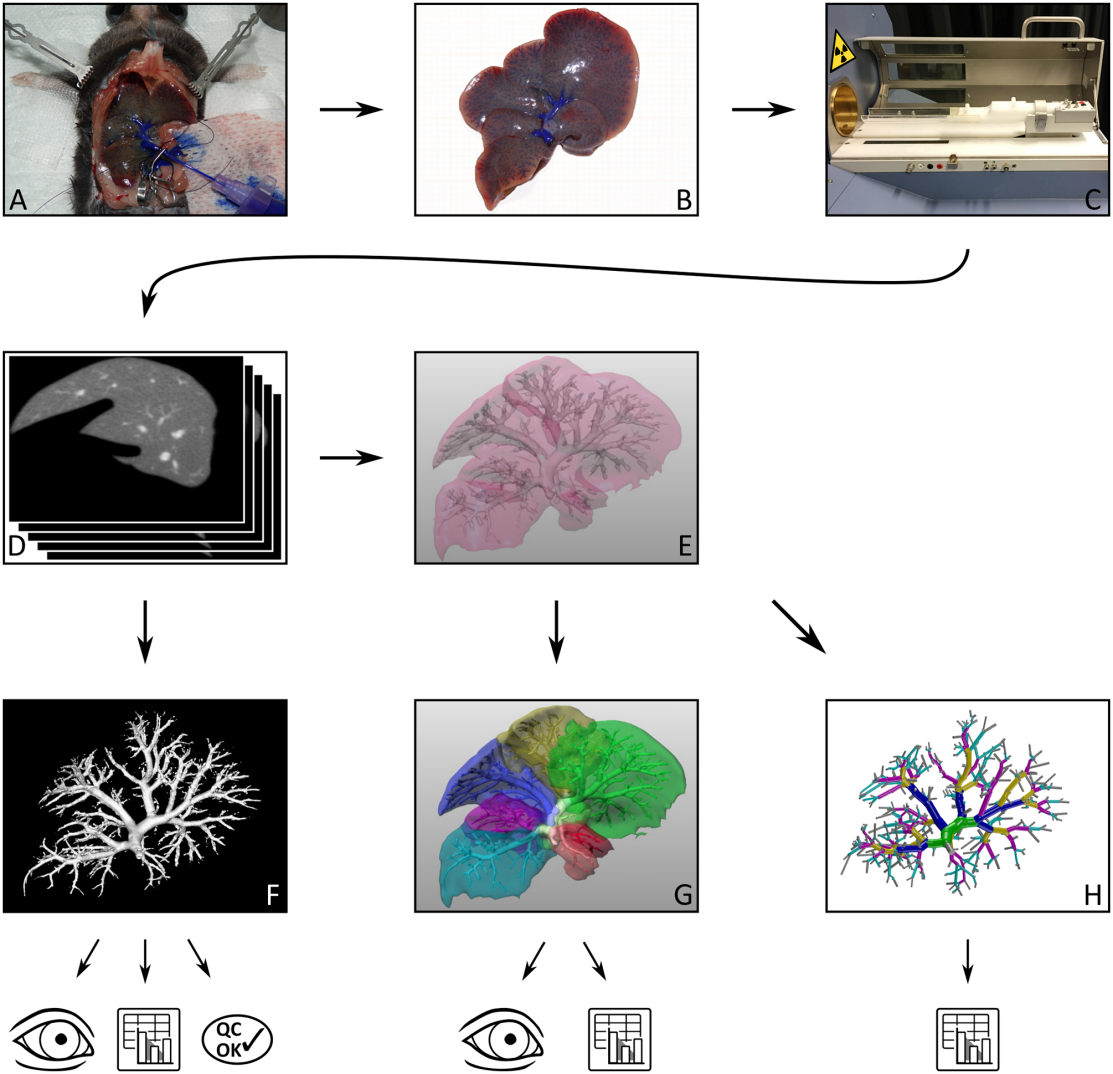
Workflow Sketch. At different time points before and after partial hepatectomy, silicone contrast compound was injected into either portal vein (shown here; naïve liver) or hepatic vein of a mouse liver (A). The liver was explanted (B) and imaged in a micro-CT scanner (C). The resulting voxel image data (D) was thresholded and visualized as a surface rendering (F), where the measurements of maximal vessel length and radius of RIL were performed interactively. From (D), the liver was segmented and the vascular system was segmented and skeletonized, resulting in a graph representation visualized in (E) along with the liver mask. Manually labeling the vascular graph according to anatomical lobes, the respective supplied territories were computed (G; territories shown in different color), permitting both qualitative visual assessment and quantitative volume measurements. The vascular graphs from (E) were converted to strictly bifurcative trees (H, here colored according to Strahler* order [21]). Based on these, automatic measurements of lengths, radii, and angles were performed. Image credits: The eye and spreadsheet icons at the bottom were adapted from https://openclipart.org/detail/216030/eye-lineart and https://openclipart.org/detail/198552/mono-spreadsheet. Photos in (B) and (C) were edited to remove irrelevant details.

The workflow of creating and scanning specimens of the livers (n = 3 animals/ time point in the PV group or the HV group) as well as subsequent image analysis is illustrated in (Fig 1).

### Experiments and Image Acquisition

#### Animals

Animal experiments were performed in male inbred C57BL/6N mice (25 g to 30 g, 8–10 weeks, Charles River, Sulzfeld, Germany). Mice were fed a laboratory diet (Ssniff Spezialdiäten GmbH, Soest, Germany) with water and mouse chow ad libitum until surgery and were kept under constant environmental conditions with a 12-h light—dark cycle. All procedures and housing of the animals were carried out according to the German Animal Welfare Legislation. Procedures involving animals were approved by Thüringer Landesamt für Verbraucherschutz, Abteilung Tiergesundheit und Tierschutz, Thüringen, Germany (Permit number: 02-122/12).

#### Partial Hepatectomy

All surgical interventions were performed under inhalation of 2% isoflurane mixed with 0.3 L/min oxygen (Isoflurane vaporizer, Sigma Delta, UK). A precise vessel-oriented, parenchyma-preserving piercing suture ligation method was used for 70% partial hepatectomy [22] in mice. In brief, after fully exposing the liver, a ligation (6–0 silk, Ethicon, US) was performed 3 mm from the main branch of the left lateral hepatic vein for removing the left lateral lobe (LLL). Next, the gallbladder was removed after ligating the cystic duct and artery (7–0 prolene, Ethicon, US). One clamp was placed roughly perpendicular to the surface of the left median lobe (LML) and the LML was removed. After removing the lobe, one piercing suture was placed to ligate the left hepatic vessels. Thereafter, the right median lobe (RML) was clamped and removed in a similar way. After resection, two piercing sutures were placed to ligate the right and median hepatic vein as well as the arterial and portal supply. Finally, the abdomen was irrigated with warm saline solution and closed with a 2-layer running suture (6–0 prolene, Ethicon, US).

After operation, animals received a subcutaneous injection of 0.05 mg/kg (body weight) buprenorphine (Temgesic, Essex Pharma GmbH, Munich, Germany) to achieve analgesia. The animals were placed on a heating pad for postoperative recovery.

In order to allow the comparison of vascular parameters of non-resected lobes before and after surgical resection and to eliminate the immediate effects of the surgery (details discussed below), a virtual resection was performed as explained below in the section “Obtaining Vascular Tree Representations”.

#### Specimen Preparation

Injection of a polymerizing silicone contrast compound (Microfil, Flow Tech Inc., Carver, US) (Fig 1A) was performed on anesthetized normal mice and mice subjected to liver resection immediately after surgery, on postoperative day (POD) 2, and on POD 7 under anesthesia (n = 3 for each time point and each group, PV and HV) [23]. Systemic heparinization was achieved by injecting heparinized saline (300 U/kg) via the penile vein and waiting for 5 min for fully systemic heparinization. After laparotomy, portal vein was cannulated with a 26-gauge heparinized catheter and flushed with heparinized saline (0.4 ml/min) using a volume-controlled perfusion device (Perfusor VI, B. Braun, Melsungen, Germany) to remove the blood from liver and to prevent blood clotting. The mice were sacrificed by exsanguination after perfusion.

Two specimen preparation methods were utilized. In the portal venous system, the silicone compound was injected into the liver via the catheter in portal vein. In the hepatic venous system, the infrahepatic inferior vena cava (IVC) was cannulated with another 26G catheter. After ligating the branches of the infrahepatic IVC and after clamping the suprahepatic IVC, the silicone compound was injected via the IVC into the hepatic venous system.

The quality of silicone injection was monitored by the naked eye under the microscope throughout the procedure. After polymerization, the specimen was explanted (Fig 1B) and weighed. Subsequently, the specimen was immersed in formalin for fixation.

#### Micro-CT Scanning

The formalin-fixed specimens were scanned by μCT (Fig 1C; Tomoscope Duo CT, CT Imaging GmbH, Erlangen, Germany). The μCT scans were performed using the scan-protocol HQD-6565-390-90 with 720 projections (approx. 1032 x 1012 pixels) during one full rotation with a scanning time of 90 s per subscan [24]. The scans resulted in *voxel image representations* (Fig 1D) of the specimens at an isotropic resolution of 70 μm.

### Image Processing and Analysis

After an initial quality check, part of the quantitative description of regeneration was obtained using the voxel image representation. Other parameters of the description were computed from an object-based representation obtained as described below. The parameters reported here reflect those parts of the vasculature resolved in the digital representations.

The vascular system was visualized in 3D (Fig 1F) based on the μCT images. For this purpose, image segmentation was performed by setting a threshold between the soft tissue intensity and the vessel intensity using Imalytics Preclinical software [25]. The resulting *3D vascular mask* was visualized using surface rendering and inspected visually to assess the injection quality. Injection and subsequent imaging was classified as successful if segmentation resulted in the visualization of an intact vascular tree without ruptured structures.

#### Circumscribed Parameters

Circumscribed parameters (maximal vessel length, in/outflow vascular radii of the right inferior portal vein (RIPV) and right inferior hepatic vein (RIPV) were determined in the right inferior lobe (RIL).

Measurement of maximal vessel length: The maximal vessel length of the right inferior portal/hepatic vein (RIPV/RIHV), i.e., the intravascular distance from root to most distant tip, was measured interactively based on the voxel images by Imalytics Preclinical software [25]. A start point marker and an end point marker were placed at the root and distal end of RIPV/RIHV in 3D-vascular mask. The path line distance inside the vessel was determined as part of computing the “Path tortuosity”.

Measurement of in/outflow vascular radius: Similarly, a start point and an end point were set at both sides of the vascular root to measure in/outflow vascular radius of the RIPV/RIHV. They were placed in the center of a vessel using a slice-based view. The vessel diameters were computed by performing automatic analysis of “Elastic sphere diameters” [26]. The radius was calculated as half the diameter.

#### Vascular Tree Representations

Vascular tree representations of the whole/remnant liver were obtained from μCT imaging data as follows:

First, a *vascular graph representation* of the vascular systems in the Microfil specimens as well as a *mask representation* of the liver (both shown in Fig 1E) and the different liver territories (Fig 1G) was obtained applying a semi-automatic procedure for clinical liver surgery planning [27;28]. This procedure consists of (a) liver segmentation, (b) vascular segmentation, (c) conversion of portal vein and hepatic vein to graph structures, (d) labeling subgraphs according to the anatomic lobes, and (e) computation of the PV-supplied or HV-drained territories based on the liver segmentation (from step a) and labeled subgraphs (from step d). Organ volumes were immediately obtained from the liver masks. Similarly, the volumes of the territories were computed based on the territory masks.

Second, the vascular graphs were converted to a *tree representation* (Fig 1H) of the vascular systems as described in [21]. This result in strictly bifurcative trees with edges represented as cylinders, i.e., each branching connects one edge to two daughter edges, each edge geometrically has straight centerlines and constant radius, in other words represents a simplification of the actual vascular segment.

#### Virtual Resection

Virtual resection was performed using HepaVision software (Fraunhofer Institute for Medical Image Computing MEVIS, Bremen, Germany). After loading the vascular graph of whole normal liver in the software, vessels which supplied/drained the remnant liver lobes in the surgical resection (RIL, RSL, CIL, and CSL) together with portal stump or partial vena cava were selected and saved as a new graph. The vessels supplying/draining the LLL, LML and RML lobe were thereby omitted from the new vascular graph. The virtual remnant liver was considered as “liver after virtual resection” and was used to eliminate any additional influence of the surgical procedure. Similarly, the subgraph for the RIL was saved separately. Virtually resected liver and RIL subgraphs were separately converted to *tree representations*, in the same way as described above for the full trees, for further separate analysis.

#### Cumulative Parameters

The tree representation of the vascular systems permits an automatic computation of different quantitative parameters describing the geometry of the vascular system, based on the methods developed for [21;29].

Total edge length and total vascular volume for a given vascular tree were computed as the sum of all lengths and volumes, respectively, of the cylinders representing vascular edges.

To characterize vascular density, vascular volume fraction was computed as the ratio of total vascular volume and parenchymal volume. Similarly, the ratio of total edge length and parenchymal volume was computed.

Given the fact that the extrahepatic part of the vascular systems could not be excluded from the vascular trees due to the technical reasons, the calculation of these parameters describing vascular density (vascular volume fraction and total edge length/hepatic volume) were based on a selected liver lobe (RIL).

#### Quantifying Regeneration

To quantify regeneration, the circumscribed and cumulative parameters were compared between different time points. Changes are reported as fold increase compared to the state immediately after surgery and as percentages of recovery compared to normal mice (i.e., before surgery).

First, vascular radii and maximal vessel lengths of the main right inferior portal/hepatic vein were measured and compared between different time points (circumscribed data for individual vessels). Next, total edge length and total vascular volume for the whole/remnant liver and separately for the RIL were evaluated (cumulative data for the vascular trees). Finally, parameters describing the vascular density, i.e., total edge length/hepatic volume and the vascular volume fraction were computed.

Moreover, the distribution of branching angles before resection and at POD 7 was compared using the similarity measure from [21], details of this approach are summarized in S1 Text.

#### Characterizing the Growth Pattern

Growth refers to a positive change in size over a period of time, in our case, includes vascular dilatation and vascular proliferation. The growth pattern was assessed with respect to isotropic expansion, defined as the increase of length by the same factor in all spatial directions.

For this comparison, the observed changes of selected descriptive parameters (see S1 Text) were compared to those changes expected in case of hypothetical isotropic expansion.

The descriptive parameters introduced above were based on the visible (i.e., digitally represented) vasculature. During regeneration, the liver increased its size. However, visibility is determined by the μCT resolution which is essentially independent of the specimen size and, in particular, independent of the time point relative to surgery. For hypothetical two specimens of the same liver before resection and at POD 7, the increase in visible vasculature would be due to two effects: (a) vascular edges exceeding the “visibility threshold” θ until POD 7 that were not yet visible before resection, and (b) growth of vascular edges already visible before resection. The latter vascular edges were denoted as “θ-visible” here to indicate that they are a subset of all visible edges. To quantify only the actual growth, the analysis was restricted to comparing parameters for the entire visible vascular trees before resection to parameters for the θ-visible part of the vascular trees at POD 7.

A precise criterion for θ-visibility (“thresholded visibility”) is described in S1 Text, along with details on how the expected changes in case of isotropic expansion of vascular parameters are were computed.

## Results

### Visualization and Quantification of Regeneration

**Fig 2:**
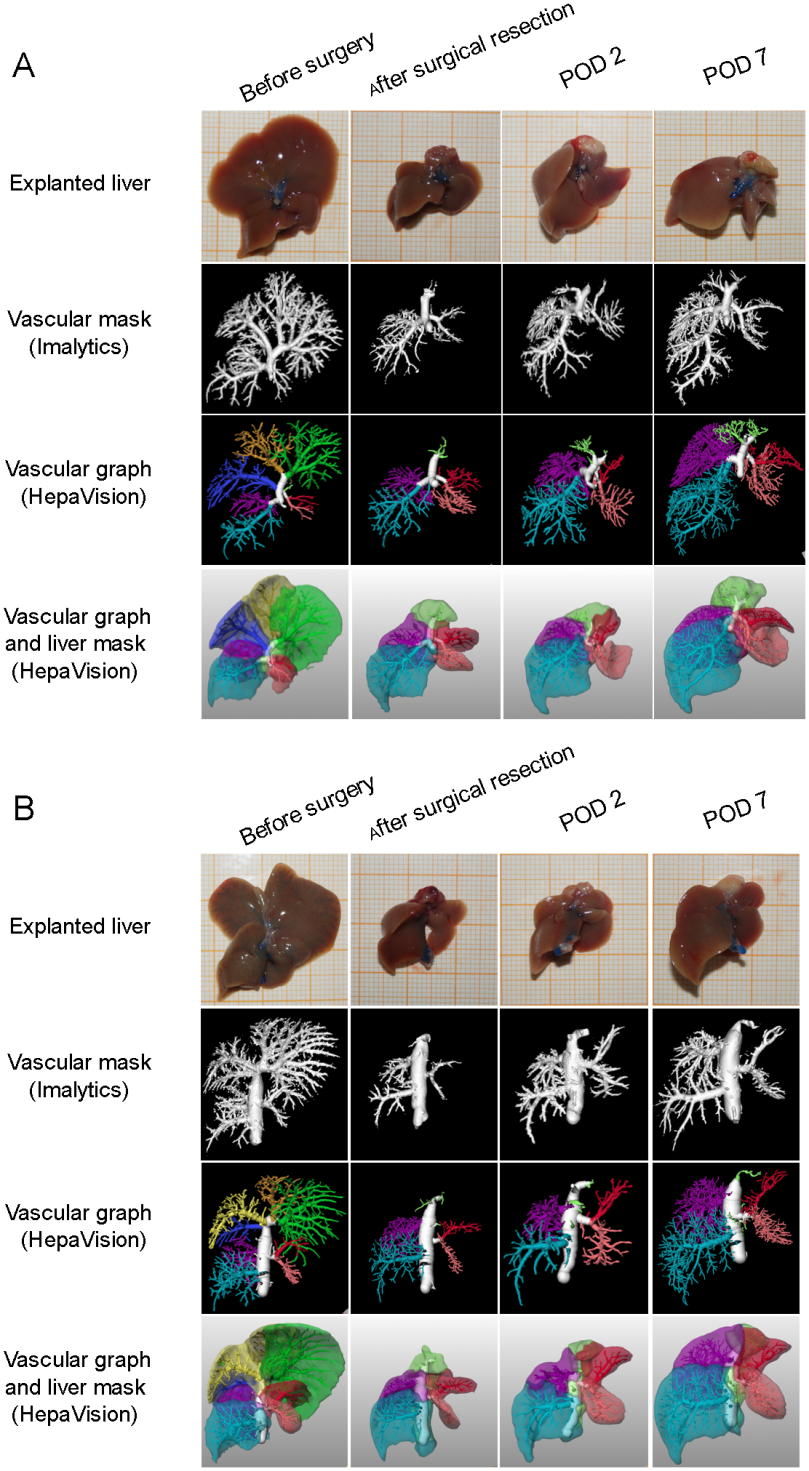
Visualization of liver regeneration. Parenchymal and vascular regeneration in the PV group (A) at indicated time points following partial hepatectomy. Left lateral lobe (visualized in green), left median lobe (visualized in yellow), and right median lobe (visualized in dark blue) were removed during PH. These lobes did not regrow during the whole regenerative procedure. Vascular regeneration in both portal and hepatic venous system appeared as elongation of the main vascular structures in combination with an out-branching of smaller vessels. Parenchymal and vascular regeneration in HV group (B) were in parallel to the growth in the PV group.

Based on the macroscopic appearance and visualizations (Fig 2), parenchymal regeneration is described qualitatively as three-dimensional growth of the remaining liver lobes. Vascular regeneration in both portal and hepatic venous system appeared to be as elongation and widening of the main vascular structures in combination with an out-branching of smaller vessels. Starting on POD 2, the vascular trees of remnant lobes elongated. By POD 7, additional small branches from the same main portal vein and hepatic vein became visible.

#### Quantification of Parenchymal Regeneration

Quantitative analysis focused on evaluation of parameters indicative of parenchymal and vascular liver regeneration at different observation time points after 70%PH.

**Fig 3:**
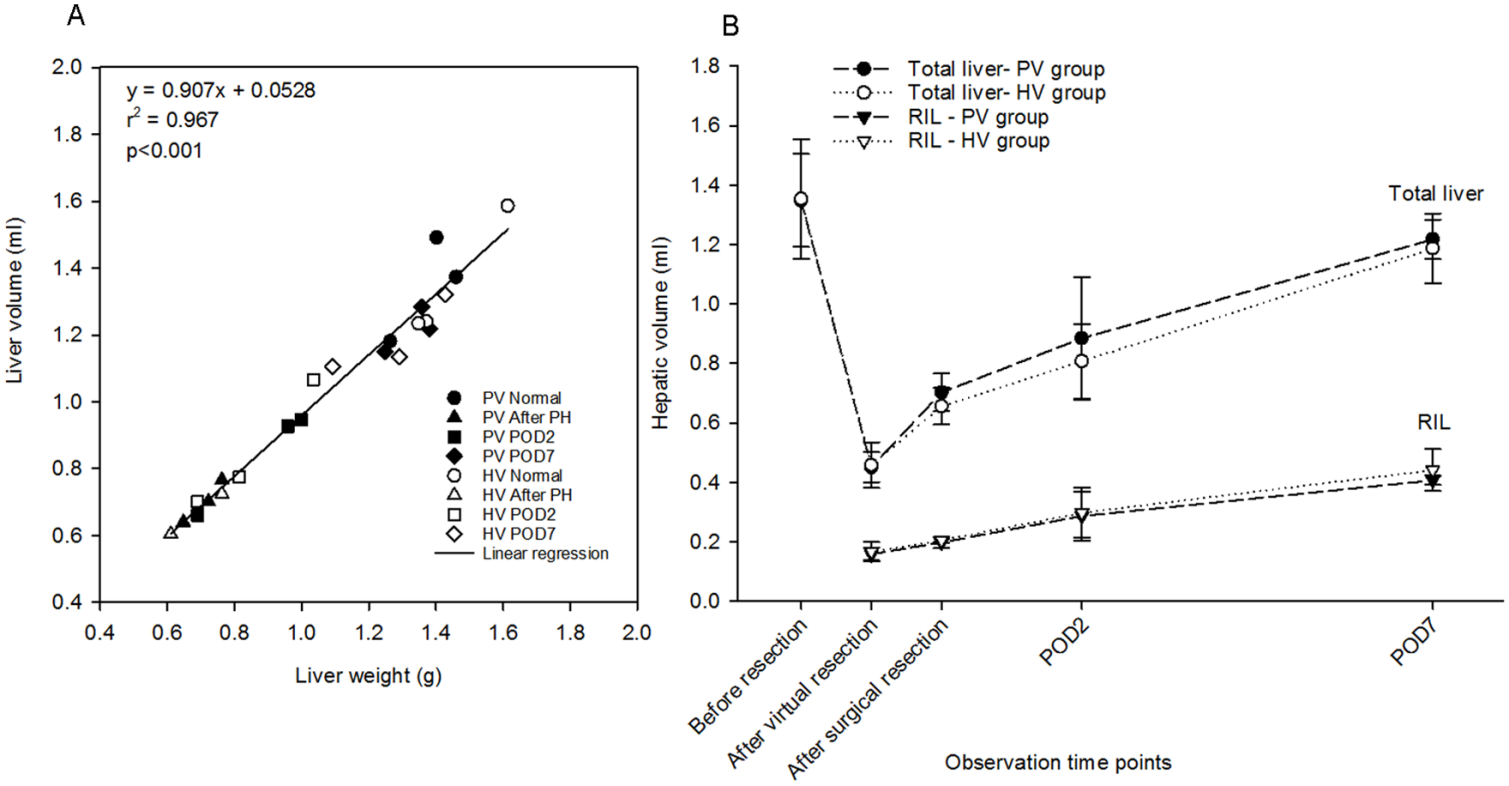
Quantification of Parenchymal Regeneration. (A) Correlation of liver weight and liver volume. Liver weight was compared to computed liver volume, resulting in the relation hepatic volume (in ml) = 0.907 × liver weight (in gram) + 0.053. These two hepatic parameters indicative of parenchymal regeneration were highly correlated (r^2^ = 0.97, p<0.001). (B) Recovery of hepatic volume of total liver and right inferior lobe within the first postoperative week (mean ± SD, n = 3/time point). In PV group, virtual resection resulted in a volume reduction from 1.35 ± 0.16 ml to 0.45 ± 0.05 ml, representing a loss of about 70% of the total hepatic volume. In contrast surgical resection caused a reduction to 0.70 ± 0.06 ml, suggesting that surgical removal of a liver lobe resulted in a stump which contributed to the volume of the remnant liver. Liver volume reached 1.22 ± 0.06 ml, representing a 90% recovery. The hepatic volume recovery was almost in parallel in the HV and PV group.

Liver weight recovery and liver volume recovery were highly correlated. The average density (± standard deviation) of the livers was 1.042 ± 0.050 g/ml, which is nearly the same as the liver density of humans (1.051 ± 0.013 g/ml) [30]. The increase of hepatic volume paralleled the increase of liver weight (Fig 3A), indicating that the increase of hepatic volume can be utilized as basis for the subsequent quantitative analysis of hepatic parenchymal regeneration.

Hepatic volume of the remnant liver (Fig 3B) was slightly lower after virtual resection compared with the volume after surgical resection. Virtual resection in the PV/HV group resulted in a decrease of mean total hepatic volume to 35% respectively 34%, whereas surgical resection resulted in a decrease of mean total hepatic volume to 48% respectively 52%. The observed volume difference between virtual resection and surgical resection may be attributed to the complete removal of the liver lobe after virtual resection. In contrast, surgical removal of a liver lobe results in a stump which contributes to the volume of the remnant liver, leading to a slightly higher remnant liver volume.

Hepatic volume after virtual resection until POD7 increased to about 90% of the starting volume and was equivalent to a 2.7/2.6-fold increase (PV/HV). Similar results in terms of fold increase were reported when studying liver weight recovery in mice [31].

The volume recovery of the RIL was very similar compared to the total liver volume recovery, indicating a similar parenchymal growth of the liver lobes compared to the whole organ.

#### Quantification of Vascular Regeneration

Assessment of vascular regeneration was based on a comparison of different parameters obtained on POD 7 and after virtual resection. This time point was chosen as the initial state to exclude immediate effects of the surgical intervention.

**Fig 4:**
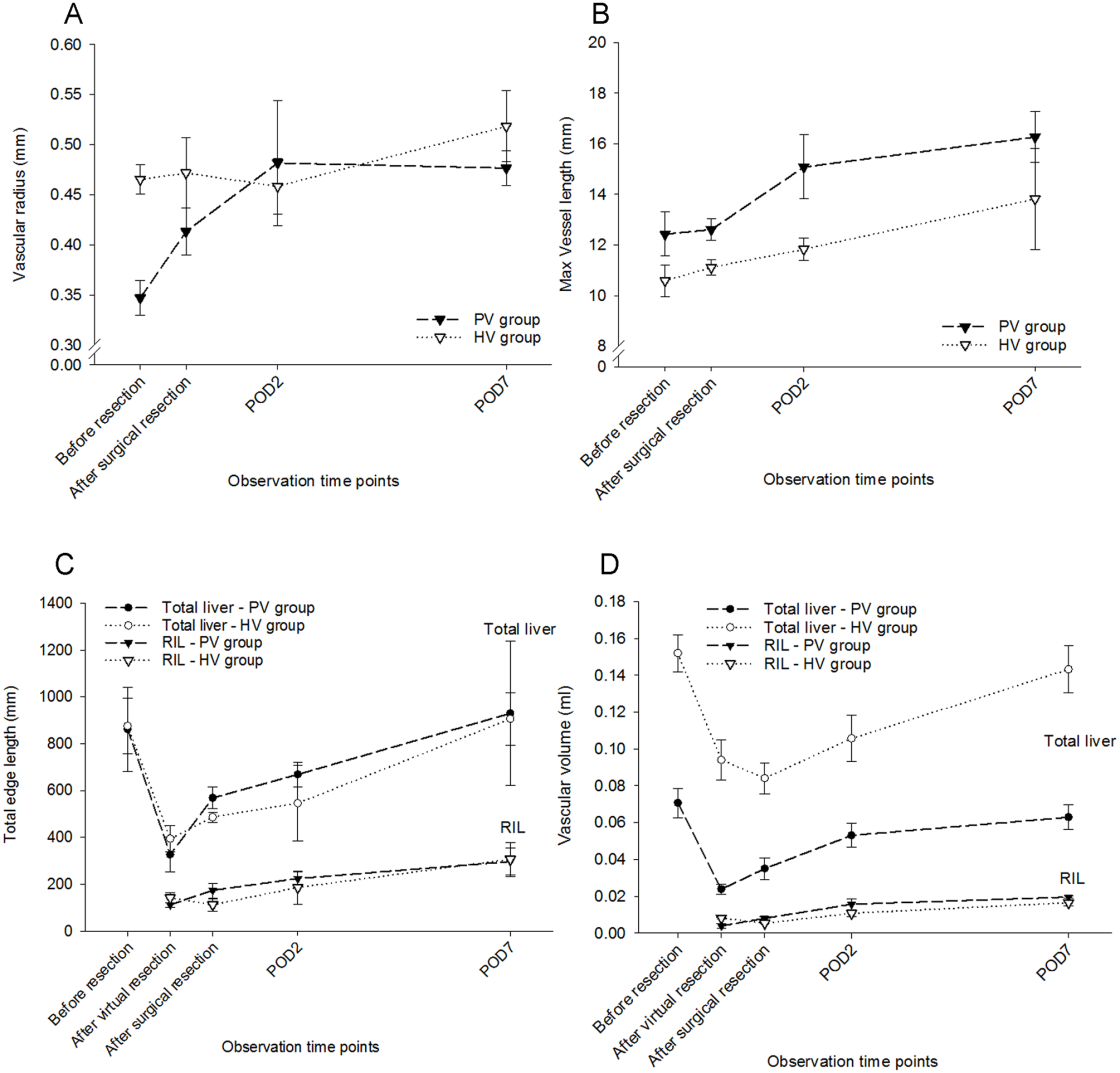
Quantification of Vascular Regeneration. Vascular radius (A) of the right inferior portal vein increased substantially until POD 2 and remained stable thereafter. In contrast, the radius of the right inferior hepatic vein remained rather similar until POD 2 and increased thereafter. However, average maximal vessel length (B) of both vascular systems increased in parallel during regeneration. Total edge length (C) in PV group reduced from 861.93 ± 179.45 ml to 326.62 ± 75.43 ml comparing before and after virtual PH, representing a loss of 62% of the total edge length. In contrast, surgical PH caused a reduction to 568.39 ± 45.94ml, suggesting an effect of portal hypertension. It reached 929.76 ± 308.88 ml, representing a 1.1-fold increase. The increase of total edge length in HV group was almost in parallel in the PV group. However, total vascular volume (D) differed between PVs and HVs. We observed an increase of total vascular volume after surgical resection compared to after virtual resection in PV group but a decrease in HV group. The increases during the regenerative process in both vascular systems were almost comparable.

Individual Vessels: Vascular radii of RIPV and RIHV (Fig 4A) increased, albeit to a different extent. Starting from mean in/outflow vascular radius of 0.35 ± 0.02 mm (PV group) and 0.47 ± 0.01 mm (HV group), radii increased by 1.3- and 1.1-fold respectively until POD 7.

When comparing the RIPV-radius after virtual resection with the radius obtained after surgical resection, a substantial difference was observed, suggesting an effect of portal hypertension. A further increase was observed when comparing the radius after surgical resection and the radius at POD 2. This increase could be the result of vascular growth but also of vascular dilatation, which cannot be discriminated based on the imaging data.

In contrast, the radius of the RIHV remained rather similar until POD 2 and increased thereafter, an observation better attributable to vascular growth.

However, average maximal vessel length of both vascular systems (Fig 4B) increased in parallel during regeneration.

Vascular Trees: In contrast to the maximal vessel length, the total edge length increased after surgical resection compared to virtual resection.

In normal mice, the total edge length of entire liver (Fig 4C) was 861.93 ± 179.45 mm in the PV group and 875.87 ± 118.87 mm in the HV group. As expected from the resected volumes after virtual resection, it decreased to 38% and 45% in the PV group and HV group.

However, total edge length was substantially higher after surgical than after virtual resection. On the one hand, this might be due to portal hypertension induced vascular dilatation caused by surgical resection (see S1 Fig). Small terminal vessel branches in both vascular systems, which were invisible in normal liver, might have been dilated and thereby became visible, which contributed to the computed total edge length. On the other hand, the vessels of the remnant stump after surgical resection were taken into account when computing the total edge length. However, the influence of the small stumps to the total edge length of the remnant liver was limited and negligible since the stumps were rather small.

The total and RIL edge length in both portal venous and hepatic venous tree recovered fully by POD 7.

Total vascular volume differed between PVs and HVs (Fig 4D). In the PV group of whole livers, volumes (0.071 ± 0.008 ml) were lower than in the HV group (0.152 ± 0.010 ml) where the volume of the inferior vena cava was also included.

Also for this parameter, we observed a difference between surgical and virtual resection. Total vascular volume in the PV group after surgical resection was slightly higher than after virtual resection. In contrast, in the HV group, total vascular volume after surgical resection was slightly lower than after virtual resection.

By POD 7, there was a 2.6-fold increase in the PV group and 1.5-fold increase in the HV group, leading to a comparable recovery (89% to 94%) within one week. Similar observations were obtained for both vascular trees of the RIL.

### Relating Parenchymal and Vascular Regeneration

#### Vascular Density

**Fig 5:**
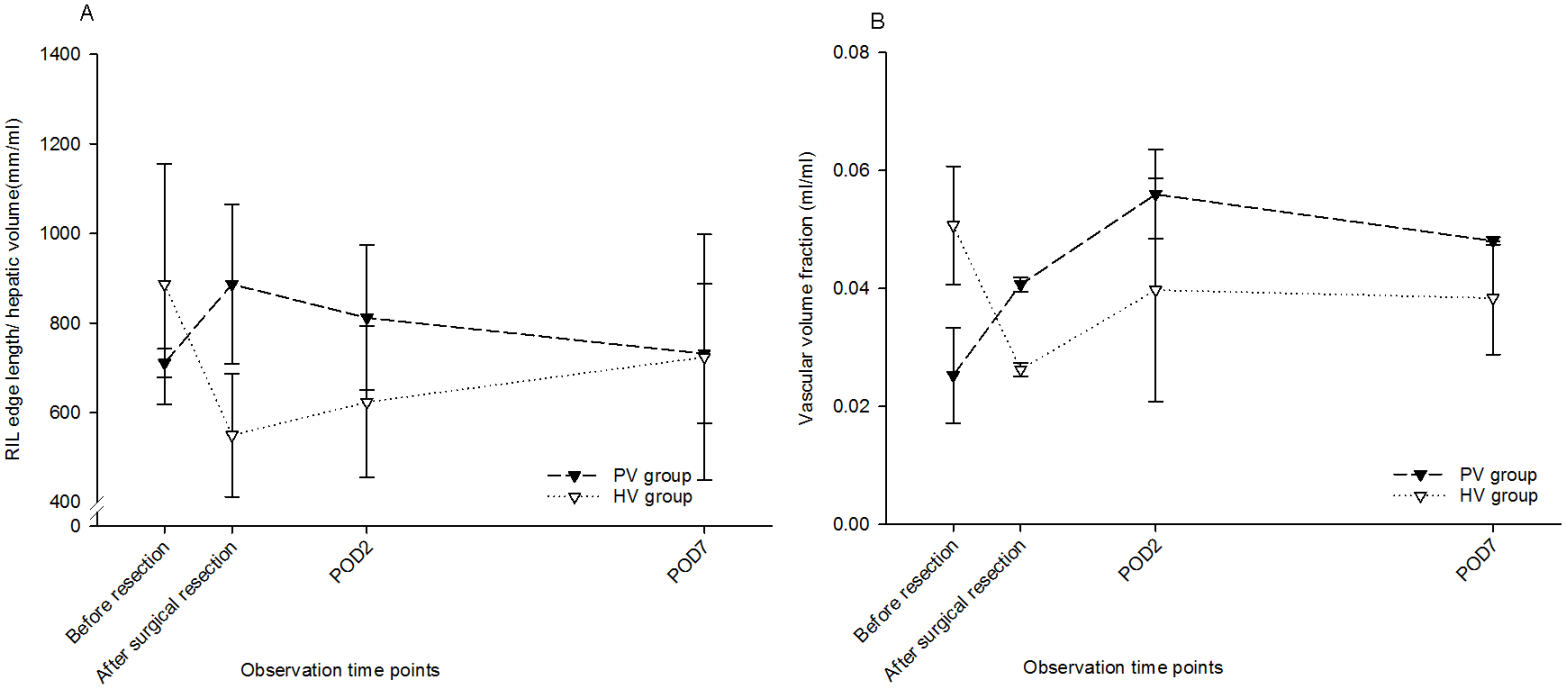
Relating Parenchymal and Vascular Regeneration in RIL. (A) RIL edge length/RIL hepatic volume. We observed an increase of RIL edge length/RIL hepatic volume in the PVs and a decrease of RIL edge length/RIL hepatic volume in the HVs after resection. This density returned to the values before surgery after 7 days’ regeneration. (B) Vascular volume fraction of RIL in PVs increased substantially when comparing the fraction before and after resection but reached its maximum on POD 2. However, the vascular volume fraction in HVs followed the same kinetic pattern as described for the HV length/RIL volume.

Similar to previously mentioned parameters, vascular density also seems to be affected by the surgery-induced portal hypertension as expected based on the edge length and vascular volume described above (see also Fig 4C and 4D). We observed an increase of PV edge length/RIL hepatic volume and a decrease of HV edge length/RIL hepatic volume after resection, possibly also an indirect effect of portal hypertension. During the course of regeneration, this density returned to the values before surgery, see Fig 5A.

In contrast, the PV volume fraction increased substantially when comparing the RIL before and after resection but reached its maximum on POD 2. This could be explained by portal hypertension still being present, while parenchymal volume is not fully recovered on POD2. The HV volume fraction followed the same kinetic pattern as described for the HV length/RIL volume, see Fig 5B.

#### Observed Growth Pattern

To characterize the observed growth pattern, relative changes of the parameters above were computed and compared.

As expected, the vascular parameters diameter and length did only increase slightly whereas the volume parameters increased substantially suggesting isotropic expansion.

**Fig 6:**
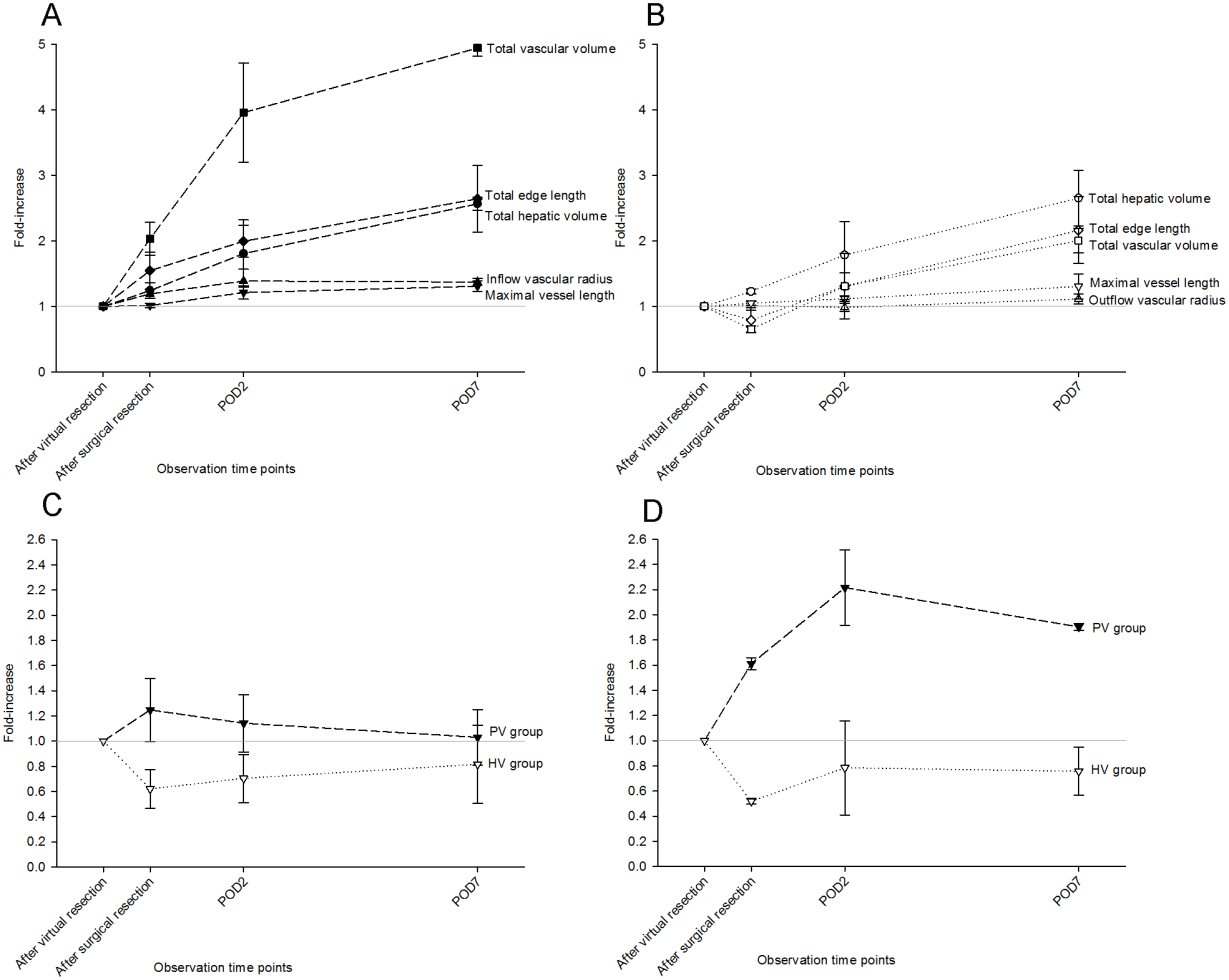
Relative changes of regenerative parameters. The relative changes of five parenchymal and vascular regenerative parameters in PV group (A) and in HV group (B) were related. After 1 week, vascular radius and maximal vessel length in PV group increased to 1.4-fold and 1.3-fold compared with after virtual resection, hepatic volume increased to 2.6-fold, total edge length and total vascular volume increased to 2.6-fold and 4.9-fold, revealing that the radius of vascular branches in the periphery increased in parallel to the radius at the inflow. The relative increases of vascular radius, maximal vessel length and hepatic volume were comparable in HV group and in PV group. However, RIL edge length and vascular volume increased not as pronounced as in PV group, to 2.2-fold and 2.0-fold respectively. This indicates that the radii in the periphery increase less than the radii at the outflow. The relative changes of these two derived parameters referred to vascular density, RIL edge length/hepatic volume (C) and vascular volume fraction of RIL (D) were compared. The increase of total edge length was much higher than the increase in hepatic volume leading to a big increase in RIL edge length/hepatic volume right after resection in the PV group. In contrast, RIL edge length/hepatic volume in the HV group had a slightly decrease after surgical resection.

In the PV group (Fig 6A), the relative increase of vascular radius and maximal vessel length of the RIL (1.4-fold and 1.3-fold) was not that obvious compared to the remaining three parameters: Hepatic volume indicative of parenchymal regeneration increased to 2.6-fold. Total edge length and total vascular volume of RIL increased to 2.6-fold and 4.9-fold, revealing that the radius of vascular branches in the periphery increased in parallel to the radius at the inflow.

Similar results were obtained when computing the relative increase of vascular radius and maximal vessel length in the HV group (1.1-fold and 1.3-fold) (Fig 6B). The relative increase of hepatic volume in the HV group (2.7-fold) was comparable to the PV group. However, there was a slight difference between the increase of RIL edge length and vascular volume in the PV and HV group. In contrast to the PV group, the increase of total edge length of RIL (2.2-fold) was higher than the increase of vascular volume (2.0-fold) in the HV group. This indicates that the radii in the periphery increased less than the radii at the outflow.

In the PV group, RIL edge length/hepatic volume (Fig 6C) increased to 1.2-fold immediately after surgical resection. Altogether, the increase of total edge length was much higher than the increase in hepatic volume leading to a big increase in RIL edge length/hepatic volume right after resection. In contrast to the PV group with a slight increase, RIL edge length/hepatic volume in the HV group had a slight decrease after surgical resection; both with a tendency to recover within the 7 observed days.

The relative changes of vascular volume fraction (Fig 6D) were different from the changes of RIL edge length/hepatic volume. Vascular volume fraction increased considerably by POD 7 in the PV group (1.9-fold). However, in the HV group, it decreased to 52% after surgical resection, recovered to 76% of normal ranges by POD 7.

These findings could be explained by portal hypertension after resection leading to a dilation of the portal vein tree, and a corresponding compression of the hepatic vein tree.

### Relation to Isotropic Expansion

For further investigating the growth pattern, a more detailed analysis was performed: branching angles were compared and changes in descriptive geometric parameters were compared to those changes expected in case of isotropic expansion. To achieve the latter comparison, the vascular datasets were thresholded to eliminate the influence of increased visibility of vasculature that would not have been visible in a hypothetical scan of the same individual at an earlier time point (θ-invisible edges).

The branching angles did not change considerably from before resection to POD 7 (see S1 Text and S2 Fig for details). The relative increases of the observed parameters differed substantially from those increases expected in case of isotropic expansion (see S1 Text and S3 Fig for details). Moreover, differences between HVs and PVs were observed. These findings are thus incompatible with isotropic expansion.

## Discussion

### Quantifying Vascular Regeneration

In this study, we quantitatively described parenchymal and vascular regeneration after PH in mice, and compared the observed hepatic regeneration to hypothetical isotropic expansion. For this purpose, we established the presented framework for high-resolution μCT imaging and qualitative and quantitative analysis of the vascular system in normal and resected mice.

Assessment of liver parenchymal regeneration based on computed volumes is a reasonable approach for several reasons: Liver weight and volume were strongly correlated (see Fig 3), the resulting density was similar to literature results [30], and the observed volume recovery within one week was comparable to previously published findings [32] for both the PV and HV group in this study.

Assessment of the vascular system based on vascular geometry provided information regarding vascular regeneration which cannot be obtained otherwise, e.g., by classical parameters such as determination of the proliferation rate of the endothelial cells.

### Comparison to Isotropic Expansion

The relative increase of parenchymal volume outweighed the relative vascular elongation, suggesting at first glance that the growth could be explained by isotropic expansion. This suspicion is further corroborated by the lack of changes in the branching angles (see S2 Fig), as only non-isotropic growth can distort bifurcations. At first glance, this suggests that regeneration resembles isotropic expansion and thus permits an easy descriptive model.

However, the more detailed investigation of the growth pattern (see S3 Fig) eliminating the influence of increased visibility, ultimately contradicted the hypothesis of isotropic expansion of liver parenchyma and vascular structures.

### Relation to Portal Hypertension

The observations above indicated that hepatic regeneration might be a more complex process influenced by many factors. One important factor is portal hypertension. Portal hypertension induced by surgical removal of liver lobes (S1 Fig) might affect these vascular parameters to different extent, not only immediately after resection but also even after 7 days.

In order to investigate immediate effects of surgery, we compared vascular parameters obtained after surgical resection to after virtual resection. We observed that portal hypertension affected the vascular diameter strongly and the vascular length slightly. These effects were more pronounced in the PV system than the HV system. Here we suggest a cautious interpretation of the respective quantitative data in the early phase of liver regeneration as the increase could be the result of vascular growth but also of vascular dilatation, which cannot be discriminated based on the imaging data. Moreover, RIL edge length/volume was also increased immediately after surgical resection. One likely reason could also be that the total edge length was enlarged due to the increase of edge visibility caused by portal hypertension-induced vascular dilatation whereas portal hypertension has limited influence to hepatic volume. In consequence, results from the virtual resection group should serve as control when assessing regeneration in terms of total edge length and total vascular volume of the regenerating remnant liver.

We also considered potentially longer-lasting effects of portal hypertension on vascular geometry. Based on two observations, we conclude that portal hypertension/perfusion may have a persisting effect on the vascular system: On the one hand, PVP has returned to normal ranges after one week (S1 Fig). On the other hand, the geometric parameters and in particular the differences between PVs and HVs on POD 7 (Fig 6) showed patterns that could be a residue of portal hypertension. However, further work is needed to determine the 3D-growth pattern of regenerating livers and to elucidate the underlying biological processes in targeted studies.

### Limitations

The presented approach using silicone injection, μCT imaging of explanted liver specimens and image analysis is useful for quantifying vascular geometries based on standard imaging techniques. It permits the assessment of differences between different post-surgical time points. Still, certain technical limitations should be taken into account when interpreting the resulting data.

First, using the explanted livers from different animals for each time point leads to the side effect of introducing inter-individual variation. Promising technologies are under development allowing repeated in-vivo assessment of the same animals [12], allowing monitoring the kinetic of vascular regeneration of an individual liver. This would permit a better comparison of vascular growth at different time points.

Second, in the HV specimens, the intrahepatic vena cava could not be excluded from silicone injection, leading to artificially large vascular volumes. When comparing between different time points for the entire liver, this mainly affected vascular volumes, whereas the influence on the other vascular geometric parameters was negligible. The analysis of the RIL as a single lobe remained unaffected by this artifact and was therefore performed. Furthermore, a slight discrepancy between physiological vascular geometry and the silicone-injected specimens might be present.

Third, image resolution in the workflow used here was limited to 70 μm, focusing the investigation on changes in the larger vasculature rather than on angiogenesis occurring at smaller length scale [33]. In the subsequent image analysis, radius and centerline position estimation led to differences between measurements in voxel image data and graph representation. This procedure is known to slightly underestimate vascular volumes [34]. The simplification from vascular graph to vascular tree again led to differences in measurements of lengths, radii, and angles. Moreover, calculating the total vascular volume as sum of vessel represented cylinder volume, overlap/gaps were ignored, introducing two more inaccuracies that only partly compensate for each other. Finally, our thresholding approach to compare the observed growth to isotropic expansion may be insufficient to eliminate the increased visibility.

Determining the relative importance of all these uncertainties on the overall quantitative results will require a detailed sensitivity analysis. This, however, is beyond the scope of the present study.

### Perspective

The workflow presented in this study is modular in the sense that it can (a) be applied to various organs and other species, (b) use other imaging modalities (e.g., other contrasting media, other and higher imaging resolution, in vivo scans, MRI), (c) employ different approaches to obtain tree representations of the vasculature, and (d) evaluate additional geometric parameters if deemed relevant. In particular, the methods used here can also be applied to (potentially smaller) specimens scanned at higher resolution to assess changes in finer vasculature, including investigating angiogenesis [6].

Quantitative descriptions of vascular regeneration and, in particular, its spatial inhomogeneity, provide useful information. It can help to identify disturbances in liver regeneration, which is a prerequisite for possibly developing treatment strategies.

Quantitative descriptions of vascular systems are of utmost importance for perfusion studies. Perfusion is, in turn, the basis for distribution of substances from the organism to the liver and in particular for pharmacokinetics. Vascular regeneration thus leads to alterations in pharmacokinetics. Quantifying vascular regeneration can be used to extend PK simulations with spatially resolved livers [35;36] towards regenerating livers. This approach could be further used to extend simulations involving lobular regeneration [37] to the scale of the entire organ. Ultimately, this could help optimizing therapy for patients undergoing liver regeneration.

Morphological assessments as needed for addressing biological questions, such as vasculogenesis, and for distinguishing vascular regeneration and vascular dilatation are under development.

This study is an important prelude to further studies on vascular regeneration. In the future, the techniques presented here can be applied in a broad range of conditions associated with the changes of vascular features. Findings could be used as input or for validation of regeneration/growth simulations including vascular structures, e.g., combining approaches like [38] and [29] or as input for simulations estimating the recovery of liver function after resection.

## Conclusion

We successfully implemented a workflow for quantifying vascular regeneration in resected mouse livers. The observed growth pattern turned out to be more complex than mere isotropic expansion. Prospectively, this quantification approach can be used for investigating hepatic vascular regeneration under different conditions.

## Supporting Information

S1 Dataset Vascular tree datasets used in the geometric analysis. Vascular trees are stored in text format, can thus be read using any text editor or imported in spreadsheets, and viewed interactively in 3D using the viewer tool from [35].
(ZIP)

S1 Fig Portal vein pressure (PVP) after 70% partial hepatectomy. PVP was measured by inserting Millar catheter into the confluence of portal vein at indicated time points. Average PVP before resection was 6.9 ± 2.4 cmH_2_O. It increased to 11.4 ± 2.9 cmH_2_O immediately after surgical resection (Data were reported in [39]). It returned to normal ranges on POD 7 (7.8 ± 0.3 cmH_2_O). This revealed that as the new vessel bed developed, the influence of portal hypertension was reduced.
(TIF)

S2 Fig Hypothetical growth of a hepatic vascular system (top row) and its visibility in a μCT scan (bottom row). Due to limited imaging resolution, only part of the actual hepatic vasculature is visible in the μCT scan. This visibility threshold for vascular segments is independent of the total size. Hence, a scan after growth may show (i) segments that were previously present and visible, (ii) segments that were previously present but not visible, and (iii) vascular segments not previously present, but now present and visible. To compare the observed growth pattern to isotropic expansion, (ii) needs to be excluded because there is no data from the earlier time point. This is achieved by a threshold θ (from below) on the radii at the later time point, restricting the analysis to θ-visible segments. This approach may also exclude parts of (iii).
(TIF)

S3 Fig Similarity of Angular Parameters. For time points Normal and POD 7, the plot shows the inter-individual similarity of the angular parameters describing bifurcations for the vascular trees of the RIL, as well as the similarity between the respective vascular trees at the two time points. The similarity measure from [21] is a value between 0 (low) and 1 (high similarity).
(TIF)

S4 Fig Comparison to Isotropic Expansion. (A) The plot shows the expected increase in case of isotropic expansion and observed relative changes for the RIL from before surgery to θ-visible parameters at POD 7. The expected values (rectangular part of the bar) are computed for the change of mean volume of the RIL from Normal to POD 7, assuming isotropic expansion. Color of the triangle showing direction of difference (blue = observed value is smaller; yellow = observed value is larger).
(TIF)

S1 Table Data evaluation of parenchymal and vascular parameters. (XLSX)

S1 Text Methods and results for assessing growth pattern. (PDF)
