# Supplementary figures and images for: Quantification of Hepatic Vascular and Parenchymal Regeneration in Mice
Source: PLoS One. 2016 Aug 5;11(8):e0160581. doi: 10.1371/journal.pone.0160581 (PMC4975469; doi:10.1371/journal.pone.0160581)

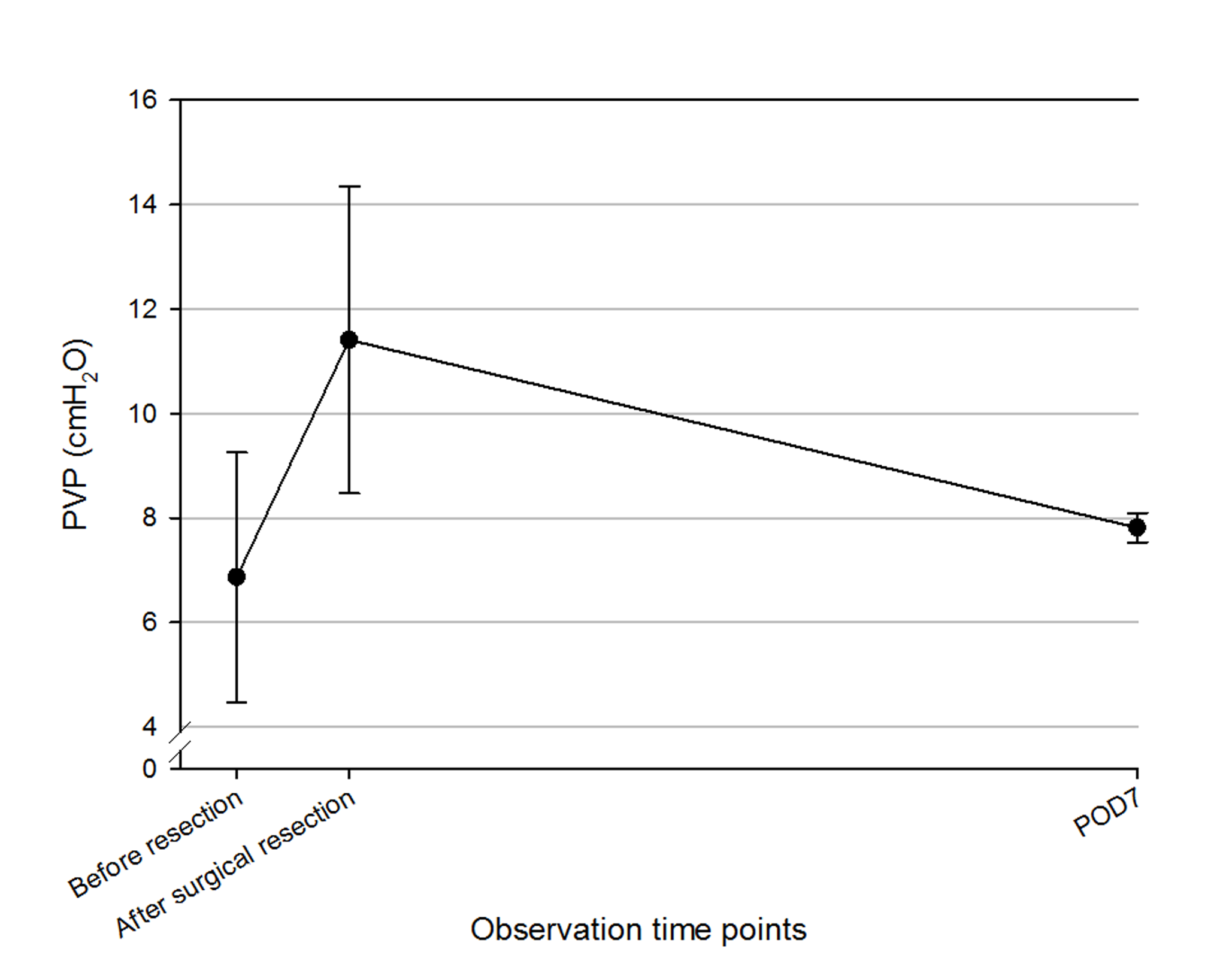

Supplement: S1 Fig — PVP was measured by inserting Millar catheter into the confluence of portal vein at indicated time points. Average PVP before resection was 6.9 ± 2.4 cmH2O. It increased to 11.4 ± 2.9 cmH2O immediately after surgical resection (Data were reported in [39]). It returned to normal ranges on POD 7 (7.8 ± 0.3 cmH2O). This revealed that as the new vessel bed developed, the influence of portal hypertension was reduced. (TIF) [file pone.0160581.s002.tif]

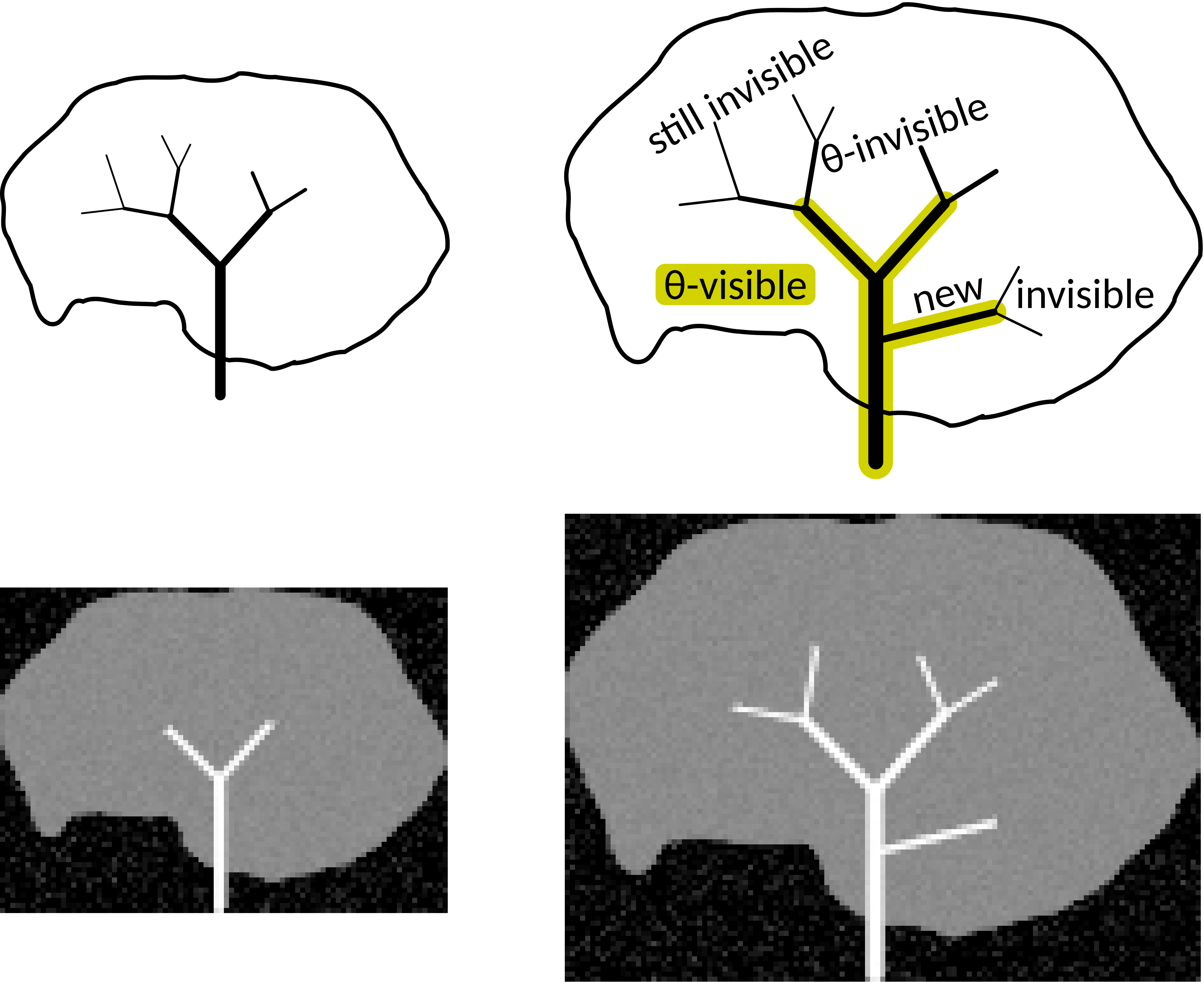

Supplement: S2 Fig — Due to limited imaging resolution, only part of the actual hepatic vasculature is visible in the μCT scan. This visibility threshold for vascular segments is independent of the total size. Hence, a scan after growth may show (i) segments that were previously present and visible, (ii) segments that were previously present but not visible, and (iii) vascular segments not previously present, but now present and visible. To compare the observed growth pattern to isotropic expansion, (ii) needs to be excluded because there is no data from the earlier time point. This is achieved by a threshold θ (from below) on the radii at the later time point, restricting the analysis to θ-visible segments. This approach may also exclude parts of (iii). (TIF) [file pone.0160581.s003.tif]

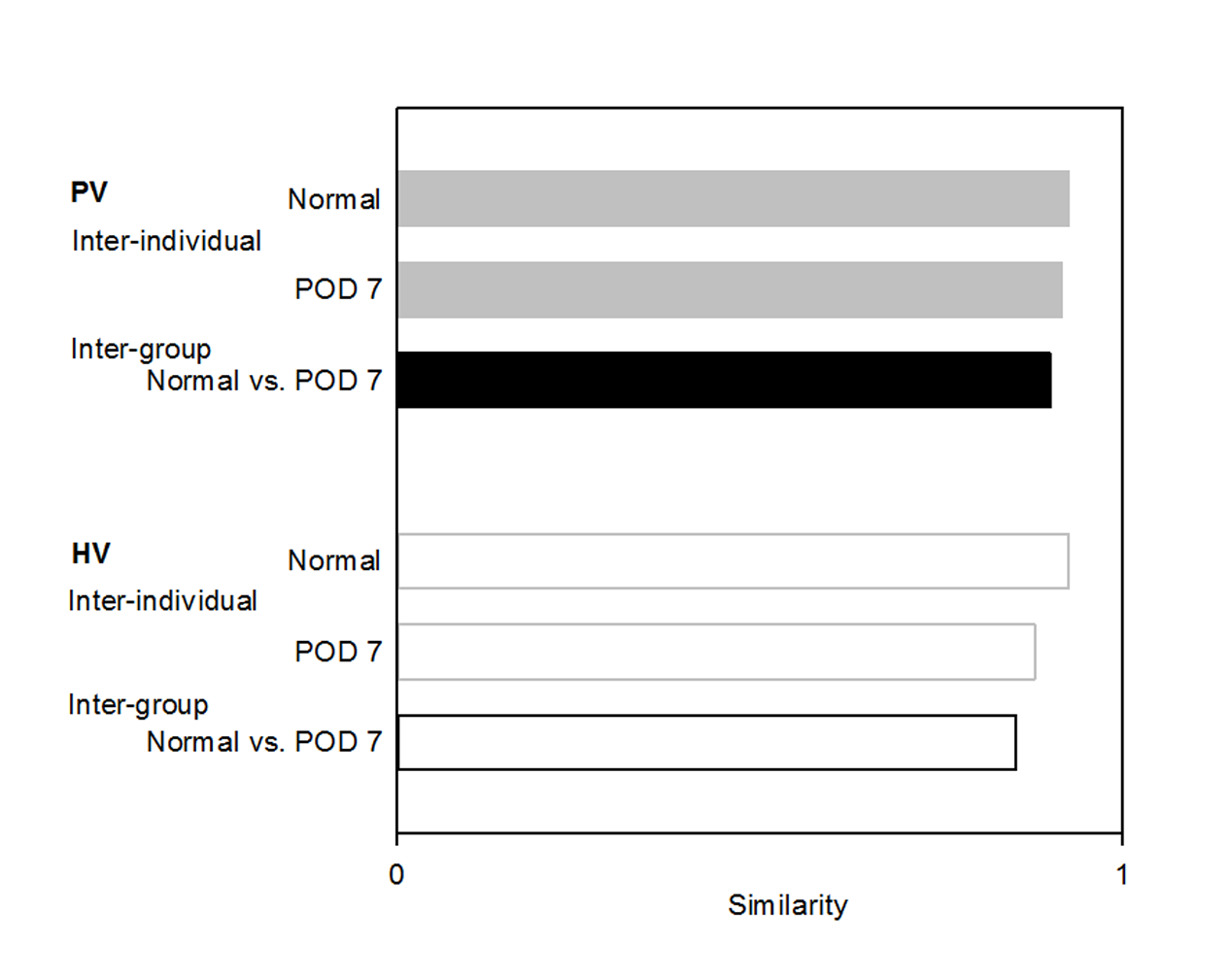

Supplement: S3 Fig — For time points Normal and POD 7, the plot shows the inter-individual similarity of the angular parameters describing bifurcations for the vascular trees of the RIL, as well as the similarity between the respective vascular trees at the two time points. The similarity measure from [21] is a value between 0 (low) and 1 (high similarity). (TIF) [file pone.0160581.s004.tif]

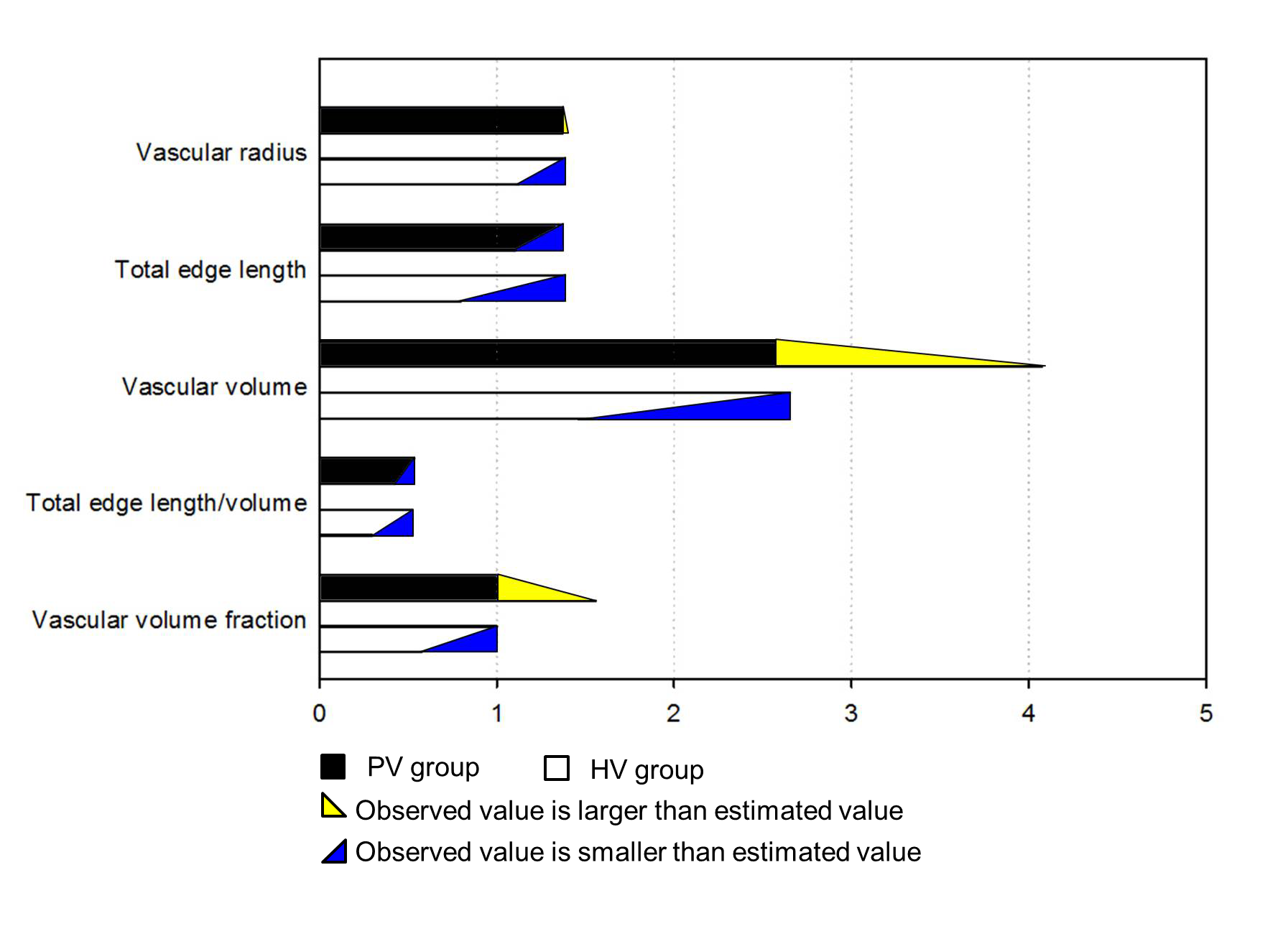

Supplement: S4 Fig — (A) The plot shows the expected increase in case of isotropic expansion and observed relative changes for the RIL from before surgery to θ-visible parameters at POD 7. The expected values (rectangular part of the bar) are computed for the change of mean volume of the RIL from Normal to POD 7, assuming isotropic expansion. Color of the triangle showing direction of difference (blue = observed value is smaller; yellow = observed value is larger). (TIF) [file pone.0160581.s005.tif]
