# Supplementary material for: Quantification of Hepatic Vascular and Parenchymal Regeneration in Mice
Source: PLoS One. 2016 Aug 5;11(8):e0160581. doi: 10.1371/journal.pone.0160581 (PMC4975469; doi:10.1371/journal.pone.0160581)
Supplement: S1 Text — (PDF) [file pone.0160581.s007.pdf]

S1 Text. Methods and Results for Assessing Growth Pattern

Chichi Xie, Lars Ole Schwen, Weiwei Wei, Andrea Schenk, Sara Zafarnia,

Felix Gremse, Uta Dahmen

Methods

Similarity of Branching Angles

The similarity of the hepatic vascular systems before resection and at POD 7 was compared in terms of angular features. For this purpose, a scalar numerical measure of similarity as introduced in [1] was used.

Each bifurcation can be fully characterized by three angles ϕ_a, ϕ_b, and ϕ_c (Fig. 1 in[1]). Based on the classical Strahler orders [2], Strahler* orders were computed for each edge in the vascular tree, as explained in [1]. Histograms of each of the angles ϕ were computed for each order and each vascular tree. Two vascular trees were classified as 'similar' in terms of one of the angles ϕ and for a fixed order if a statistical two-sample Kolmogorov–Smirnov (KS) test [3] failed to detect a significant ($p < 0.05$, [4]) difference between the respective histograms. The overall angle similarity at a given observation time point, e.g., PV Normal, was then computed by comparing, for each angle ϕ and each order, each distinct pair of PV trees, and computing the fraction of 'similar' comparisons. Finally, the mean (over all angles ϕ) of the weighted averages (over the orders; weighted according to the number of edges with the given order) of these fractions was evaluated. This resulted in a number between 0 (no similarity) and 1 (high similarity).

For a single group, this indicates intra-individual similarity. The similarity of two groups of vascular trees, e.g., PV Normal vs. POD 7, was computed in analogy to the procedure described above by comparing each normal PV to each PV at POD 7. Finally, an average over the three angles was computed. Detailed formulas for this quantification of similarity are given in [1].

Characterizing the Growth Pattern

For a comparison of the observed growth pattern to hypothetical isotropic expansion, it is necessary to exclude an effect of increased visibility.

The descriptive parameters introduced in the present study were based on the visible (i.e., digitally represented) vasculature. During regeneration, the liver increased its size. However, visibility is determined by the µCT resolution which is essentially independent of the specimen size and, in particular, independent of the time point relative to surgery. For hypothetical two specimens of the same liver before resection and at POD 7, the increase in visible vasculature would be due to two effects: (a)

vascular edges exceeding the "visibility threshold" θ until POD 7 that were not yet visible before resection, and (b) growth of vascular edges already visible before resection. The latter vascular edges were denoted as "θ-visible" here to indicate that they are a subset of all visible edges. To quantify only the actual growth, the analysis was restricted to comparing parameters for the entire visible vascular trees before resection to parameters for the θ-visible part of the vascular trees at POD 7.
Here, a precise criterion for θ-visibility ("thresholded visibility") is described, as well as the computation of expected changes in case of isotropic expansion of vascular parameters.

Thresholding for Quantitative Comparison

To define θ-visibility, a threshold θ for vascular radii was used. Generally, volume scales with the third power of length. Under the assumption of isotropic expansion, a relative change in length thus corresponds to the cubic root of the relative change in volume (see Fig. S2).
Conversely and specifically for the data at hand: if ΔV denotes the relative volume change of the RIL from before resection to POD 7, the expected relative change Δd in length under the assumption of isotropic expansion is $\Delta d = \Delta V^{1/3}$. So a threshold θ was chosen as θ=Δd times the minimal radius present in the vascular tree on POD 7. Under the assumption of isotropic expansion, any edges of radius ≥ θ would already have been visible in a hypothetical specimen of the same liver created before surgery. Thus, only edges of radius ≥ θ were used for further analysis.

Determining Parameters for Quantitative Comparison

To compare the vascular growth pattern to hypothetical isotropic expansion, part of the geometric parameters were selected. The criterion for selection was that the respective parameter could be evaluated restricted to the θ-visible edges on POD 7. These parameters were (a) in/outflow vascular radius, (b) total vascular length, (c) total vascular volume, (d) total vascular length/volume, and (e) vascular volume fraction.
In/outflow vascular radius of the RIL is always θ-visible since it was measured at the root of the vessel, so no new determination was needed for POD 7. The other parameters are affected by θ-invisibility and were thus re-evaluated restricted to θ-visible edges on POD 7 ("observed values").

Estimating the Effect of Isotropic Expansion

Based on ΔV, defined above as the relative volume change of the RIL from before resection to POD 7, “expected values” were obtained by scaling the parameter from before resection by appropriate scaling factors. These scaling factors are the relative change in length ($\Delta d=\Delta V^{1/3}$, see above) to the power of the dimension of the respective parameter, i.e.,

- $\Delta V^{1/3}$ for in/outflow vascular radius and total vascular length,
- $\Delta V^{3/3}=\Delta V$ for total vascular volume,
- $\Delta V^{-2/3}$ for total vascular length/volume, and
- $\Delta V^{0/3}=1$ for the vascular volume fraction (i.e., the latter should remain constant).

Finally, observed and expected values were compared to estimate the extent of growth.

Comparing Angular Features

Further insight into the growth pattern was gained by quantifying changes in angular features.

Purely isotropic expansion does not change any angles at bifurcations. Given that specimens before resection and at POD 7 were obtained from different animals, no 1:1 comparison was possible. Instead, the similarity of branching angles between the two time points was compared to the intraindividual similarity at the separate time points. If those similarities were in the same range, this was used as an indicator supporting the hypothesis of purely isotropic growth.

Results

Similarity of Branching Angles

Branching angles at the bifurcations (see Fig.S3) exhibited a high similarity among the normal livers, 0.926 in both the PV and the HV group. These similarities are even larger than previous results for entire mouse livers (0.841 vs. 0.710) as reported (Fig. 2 in [5]), and show a slightly higher similarity among PVs than for HVs also in our data. Among the livers at POD 7, lower similarities of 0.916 and 0.879 (among the PV and HV groups, respectively) were observed. In both cases, the tendency of PVs being more similar than HVs in terms of branching angles could be confirmed, (see [5] for results in mice and [1] in humans). The similarity of normal PVs and those at POD 7 was 0.900 and thus not substantially lower than at the separate time points (0.853

for HVs), showing that branching angles do not change considerably during regeneration.

Comparison of the Observed Growth Pattern to Isotropic Expansion

Based on the data above, the increase of parenchymal volume in the RIL was 2.6-fold (PV group; 2.7-fold for the HV group; see Fig.3-B, whereas the increase of the maximal vessel length was 1.3-fold. In case of isotropic expansion, the 2.6-fold volume increase would correspond to a 1.4-fold increase of lengths and radii. This, however, does not take into account the visibility threshold due to fixed imaging resolution.

The observed relative increase in the parameters describing the vascular system before resection and at POD 7 were compared to the expected increase in case of isotropic expansion, see Fig. S4.

In the PV cases, the increase of inflow vascular radius almost perfectly matched the expected increase in case of isotropic expansion. Total edge length and total edge length/hepatic volume were about 20% lower than expected in case of isotropic expansion, whereas the vascular volume and the vascular volume fraction were about 60% larger. This indicates that the vascular radii in the periphery increased a lot more than expected. In the HV cases, the outflow vascular radius was about 20% lower than the expected increase in case of isotropic expansion, the remaining parameters (total edge length, total edge length/hepatic volume, vascular volume, vascular volume fraction) were about 40% lower than the expected increase in case of isotropic expansion. This could indicate that HVs generally grow/regenerate slower than PVs.

In summary, the changes in the vascular systems, combined with differences between HVs and PVs, contradict the hypothesis of isotropic expansion of liver parenchyma and vascular structures.

References

1. Schwen LO, Preusser T. Analysis and algorithmic generation of hepatic vascular systems. International journal of hepatology. 2012;2012:357687. Epub 2012/10/12. doi: 10.1155/2012/357687. PubMed PMID: 23056953; PubMed Central PMCID: PMC3463918.

2. Arthur NS. Quantitative analysis of watershed geomorphology. Transactions, American Geophysical Union. 1957;38(6):913-20. doi: 10.1029/TR038i006p00913.

3. Dudewicz E, Mishra S. Modern Mathematical Statistics: Wiley; 1988.

4. Stigler S. Fisher and the 5% level. Chance. 2008;21(4):12. doi: 10.1007/s00144-008-0033-3.

5. Schwen LO, Wei W, Gremse F, Ehling J, Wang L, Dahmen U, et al. Algorithmically generated

rodent hepatic vascular trees in arbitrary detail. Journal of theoretical biology. 2015;365:289-300. Epub 2014/12/03. doi: 10.1016/j.jtbi.2014.10.026. PubMed PMID: 25451523.
